# The Effect of Antioxidants on Sperm Quality Parameters and Pregnancy Rates for Idiopathic Male Infertility: A Network Meta-Analysis of Randomized Controlled Trials

**DOI:** 10.3389/fendo.2022.810242

**Published:** 2022-02-21

**Authors:** Kun-peng Li, Xue-song Yang, Tao Wu

**Affiliations:** Department of Urology, Affiliated Hospital of North Sichuan Medical College, Nanchong, China

**Keywords:** antioxidants, idiopathic male infertility, sperm quality parameters, pregnancy rate, network meta-analysis, randomized controlled trials

## Abstract

**Purpose:**

Male infertility is a global public health issue recognized by the WHO. Recently, antioxidants are increasingly used to treat idiopathic male infertility. However, the lack of available evidence has led to the inability to rank the effects of antioxidants on the sperm quality parameters and pregnancy rate of infertile men. This network meta-analysis studied the effects of different antioxidants on the sperm quality and pregnancy rate of idiopathic male infertility.

**Methods:**

We searched PubMed, Embase, Web of Science, and Cochrane Library databases for randomized controlled trials (RCTs). The weighted mean difference (WMD) and odds ratio (OR) were applied for the comparison of continuous and dichotomous variables, respectively, with 95% CIs. The outcomes were sperm motility, sperm concentration, sperm morphology, and pregnancy rate.

**Results:**

A total of 23 RCTs with 1,917 patients and 10 kids of antioxidants were included. l-Carnitine, l-carnitine+l-acetylcarnitine, coenzyme-Q10, ω-3 fatty acid, and selenium were more efficacious than placebo in sperm quality parameters. l-Carnitine was ranked first in sperm motility and sperm morphology (WMD 6.52% [95% CI: 2.55% to 10.05%], WMD 4.96% [0.20% to 9.73%]). ω-3 fatty acid was ranked first in sperm concentration (WMD 9.89 × 10^6^/ml, [95% CI: 7.01 to 12.77 × 10^6^/ml]). In terms of pregnancy rate, there was no significant effect as compared with placebo.

**Conclusions:**

l-Carnitine was ranked first in sperm motility and sperm morphology. ω-3 fatty acid was ranked first in sperm concentration. Coenzyme-Q10 had better effective treatment on sperm motility and concentration. Furthermore, high-quality RCTs with adequate sample sizes should be conducted to compare the outcomes of different antioxidants.

## Introduction

Increasing evidence indicates that the incidence of infertility has gradually increased over the past decades. Infertility affects about 15% of couples globally (1). Male factors are estimated to be present in about 50% of cases, and 20% of cases have common contributing female factors (2, 3). Although the current research on the causes of male infertility has made great progress, idiopathic male infertility is still a challenging condition to diagnose and manage (2). Unlike unexplained male infertility (UMI) with normal sperm parameters, the patients diagnosed with idiopathic male infertility have the presence of altered sperm characteristics without an identifiable cause. We have to rule out the cause of female factor infertility (4, 5).

Overwhelming evidence suggests that oxidative stress (OS) plays a vital role in the etiology of male infertility (6). OS is an imbalance between reactive oxygen species (ROS) and protective antioxidants (7). OS could lead to abnormal sperm parameters and high levels of sperm deoxyribonucleic acid fragmentation (8). Recently, the concept “male oxidative stress infertility” (MOSI) has been proposed and pointed out that about 30%–80% of infertile men have increased ROS in sperm, which affected about 37.2 million infertile men (4). Therefore, using antioxidants to combat excessive OS in the treatment of infertility is a potential option. Several studies have reported the beneficial effects of oral antioxidants on sperm parameters. Supplementing exogenous l-carnitine, l-carnitine+l-acetylcarnitine, coenzyme-Q10, ω-3 fatty acid, selenium, zinc, vitamin E, and vitamin C has beneficial effects on sperm parameters. However, there were limited data available on pregnancy rates for interventions (9, 10–13).

Double-blind randomized controlled trials (RCTs) of direct comparison between drugs can provide clear evidence on health technology profiles. However, the high cost and a large number of patients could limit their development (14, 15). Network meta-analysis (NMA) allows the comparison of more interventions simultaneously, even if they are not directly compared by clinical trials. Compared with pairwise meta-analyses, the NMA could facilitate clinicians to mold interventions according to their choice to achieve desired outcomes in idiopathic male infertility (16).

There have been many RCTs of antioxidants versus placebo, which have demonstrated their effectiveness. However, there have been few head-to-head trials of one kind of antioxidant vs. another. So the relative efficacy of available treatment options for infertility is unclear. There are several parameters involved in the evaluation of sperm quality. Furthermore, it is uncertain, which antioxidants have the best effect on a certain parameter of sperm. Last, many articles also did not report the effect of antioxidants on pregnancy rates. So we conducted an NMA to resolve this uncertainty and found the most effective drug for male infertility.

## Methods

### Search Strategy

This systematic review and meta-analysis was conducted in accordance with the Preferred Reporting Items for Systematic Reviews and Meta-Analyses (PRISMA) statement (17). We conducted a systematic electronic literature search in February 2021 in PubMed, Embase, Web of Science, and Cochrane Library databases. The search was limited to English. We used medical subject heading (MeSH and Emtree) terms combined with Boolean logical operators (for searching strategies, see **Table S1**).

### Inclusion and Exclusion Criteria

Studies were included if they met the following criteria: 1) only RCT studies; 2) idiopathic infertility was diagnosed; 3) the patients have sperm parameter abnormalities; and 4) the primary outcomes have to include sperm motility, sperm concentration, sperm morphology, pregnancy rate, alone or in combination. Studies were excluded if they met the following criteria: 1) case–control, cross-sectional, or retrospective studies; 2) men with varicocele or other fertility-related diseases; 3) men with drug interventions; and 4) combined use of different types of antioxidants.

### Data Extraction

Two independent reviewers extracted the following data. Data extracted for the individual study included 1) general information related to the manuscript: first author, year of publication, and country. 2) Characteristics of the population: sample size, groups, age, and diagnosis. 3) Type of treatment, duration, and dosage. 4) Primary outcomes: sperm motility, sperm concentration, sperm morphology, and pregnancy rate. In cases that have multiple visits between these intervals, the longest one would be selected. We tried to make the included articles have similar characteristics to ensure the optimal transitivity of the NMA (18). Any dispute was resolved by consensus or consultation with a third reviewer.

### Quality Assessment

The quality of RCTs was evaluated according to the Cochrane Collaboration’s tool (19), including random sequence generation, allocation concealment, blinding of participants, personnel and outcome assessment, incomplete outcome data, selective reporting, and other sources of bias.

### Statistical Analysis

All the analyses were performed using Stata statistical package version 14.1 software; all statistical analyses were conducted to calculate the weighted mean difference (WMD) for continuous data and odds ratio (OR) for dichotomous variables, together with the corresponding 95% CIs. For the traditional pairwise meta-analysis, the Mantel–Haenszel random-effects model was used (20). The heterogeneity in each pairwise comparison used I^2^ statistics and was judged as low (<25%), moderate (25%–75%), or high (>75%). p-Values of <0.05 were regarded as statistically significant (21). The random-effects NMA was conducted as a frequentist approach (22). The model inconsistency must be evaluated because the presence of inconsistency is often regarded as a statistical manifestation of intransitivity (18). Both consistent and inconsistent models were analyzed and compared. Furthermore, the design-by-treatment interaction model and loop consistency were used to assess the inconsistency in the NMA. The loop-specific approach estimated inconsistency factors and their 95% CIs within every closed triangular or quadratic loop (23–25). The relative ranking of treatments was done using the surface under the cumulative ranking (SUCRA). The ranks for each outcome were also presented (26, 27). Additionally, publication bias of NMA and potential small-study effects were tested for using a comparison-adjusted funnel plot (28). Multiple sensitivity analyses were performed to address the robustness of our findings, including 1) exclusion of studies with a significant risk of bias, 2) exclusion of studies published before 2002, and 3) exclusion of sample size of less than 30.

## Results

### Literature Search Results and Characteristics of Included Randomized Controlled Trials

**Figure 1:**
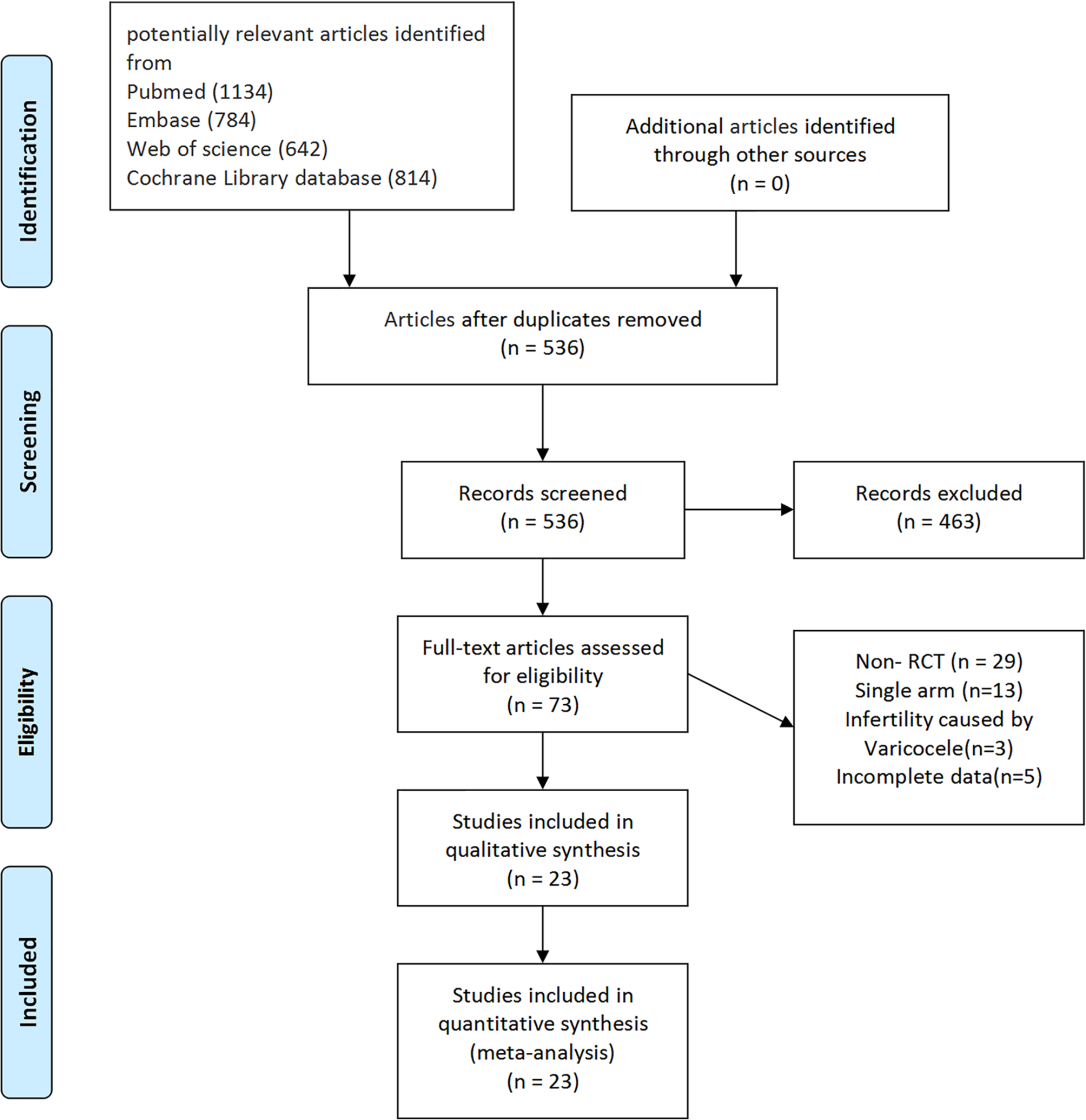
PRISMA flowchart of study selection. PRISMA, Preferred Reporting Items for Systematic Reviews and Meta-Analyses.

**Figure 2:**
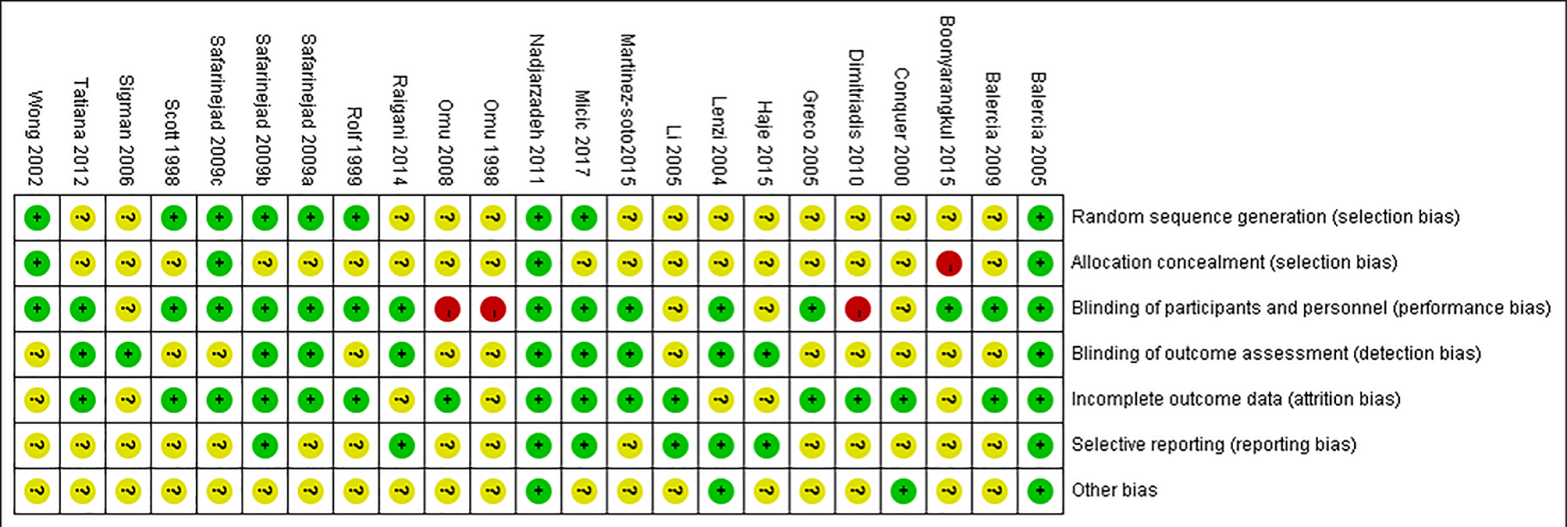
Risk of bias assessment.

According to the literature search and the inclusion criteria, we included 1,917 patients in 23 studies for meta-analysis (29–51) (**Figure 1**). The details are in **Table 1**. Those studies were published from 1998 to 2017 and evaluated 10 antioxidants including l-carnitine, l-carnitine+l-acetylcarnitine, coenzyme-Q10, ω-3 fatty acid, selenium, zinc, vitamin E+vitamin C, folic, and *N*-acetyl-cysteine. All the patients were diagnosed with idiopathic infertility with abnormal sperm parameters. Most studies included a two-arm study design, but 4 studies included a three-arm design. Of 23 studies, 2 were rated as low risk and 4 studies as high risk (**Figure 2**).

**Table 1:** Characteristics of included trials.

Reference	Site of study	Type of RCTs	Age (years)	Intervention (n)	Duration	Outcome	Population studied
Haje 2015 (29)	UK	Double-blind	37.5 ± 2.46	LC 1 g (n = 34)	3 months	①②③④	Repeated exhibition of iOA without detectable cause (iOA)
				PL (n = 29) per day			
Balercia 2005 (30)	Italy	Double-blind	30 (24–38)	LC 3 g (n = 15)	6 months	①②③④	Infertile men with iA and infertility > 2 years
				LC 3 g+LAC 1 g (n = 14)			Sperm count > 20 × 10^6^/ml, sperm motility < 50%, normal sperm morphological features > 30%
				PL (n = 15)			
Dimitriadis 2010 (31)	Japan	Single-blind	Unclear	LC 1 g (n = 26)	12 weeks	①②	Infertile men with iOA
				PL (n = 22) per day			
Lenzi 2004 (32)	Italy	Double-blind	20–40	LC 2 g+LAC 1 g (n = 30)	3 months	①②③④	Infertile men with iOAT and infertility > 2 years
				PL (n = 26) per day			
Micic 2017 (33)	Serbia	Double-blind	Unclear	LC 2 g+LAC 1 g (n = 125)	3 months	①②	Infertile men with iOA They have difficulty in conceiving > 12 months
				PL (n = 50) per day			
Sigman 2006 (34)	USA	Double-blind	LAC+LC: 36.2 ± 1.7 PL: 35.3 ± 2.5	LC 2 g+LAC 1 g (n = 12)	24 weeks	④	Infertile men with iA and at least 6 months’ duration. Sperm concentration of at least 5 million sperm/ml. Sperm motility of 10% to 50%
				PL (n = 9) per day			
Balercia 2009 (35)	Italy	Double-blind	32 (27–39)	Q10 200 mg (n = 30)	6 months	①②④	Infertile men with iA, infertility > 2 years, sperm count > 20 × 10^6^/ml, sperm motility < 50%, normal morphology > 30%
				PL (n = 30) per day			
Safarinejad 2009a (36)	Iran	Double-blind	Q10: 28 ± 9	Q10 300 mg (n = 98)	6 months	①②③④	Infertile men with iOA and infertility > 2 years
			PL: 28 ± 10	PL (n = 96) per day			
Nadjarzadeh 2011 (37)	Iran	Double-blind	Q10: 34.17 ± 4.52;	Q10 200 mg (n = 30)	3 months	①②③④	Infertile men with iOAT and have tried for pregnancy for >1 year of unprotected intercourse
			PL: 34.67 ± 6.69	PL (n = 24) per day			
Rolf 1999 (38)	Germany	Double-blind	Vitamin C+E: 36.1 ± 5.0	Vitamin C 1 g+E 0.8 g (n = 15)	8 weeks	①②③④	Men with infertility for over 1 year Infertile men with iA or iOA
			PL: 35.2 ± 4.8	PL (n = 16) per day			
Greco 2005 (39)	France	Double-blind	unclear	Vitamin C 1 g+E 1 g (n = 32)	2 months	①②③	Idiopathic infertility, a presence of fragmented DNA ≥ 15% of ejaculated spermatozoa
				PL (n = 32) per day			
Li 2005 (40)	China	Double-blind	LC+LAC: 30 ± 5.5	LC 200 mg+LAC 100 mg (n = 85)	3 months	④	Infertile men with iOAT, fertility medication must be stopped 2 weeks before
			VitaminC+E: 32 ± 3.5	Vitamin C 200 mg+E 200 mg (n = 53) per day			
Conquer 2000 (41)	Canada	Double-blind	DHA: 38.3	DHA 400 mg (n = 9)	3 months	①②	Infertile men with iA, sperm motility < 50%
			PL: 35.2	PL (n = 9) per day			
Safarinejad 2009b (42)	Iran	Double-blind	ω-3: 32 ± 9	EPA 1.12 g+DHA 0.72 g (n = 106)	32 weeks	①②③	Infertile men with iOAT, infertility > 2 years
			PL: 32 ± 10	PL (n = 105) per day			
Martinezsoto 2010 (43)	Spain	Double-blind	ω-3: 35 ± 0.8	EPA 135 mg+DHA 1 g (n = 106)	10 weeks	①②③	Idiopathic male infertility suffering from factor infertility, according to the WHO, and they were undergoing infertility evaluation
			PL: 35.6 ± 1.0	PL (n = 105) per day			
Scott 1998 (44)	UK	Double-blind	Se: 32.6 ± 1.1	Se 100 µg (n = 16)	3 months	①②	Infertile men with iA
			PL: 32.9 ± 1.5	PL (n = 18) per day			
Safarinejad 2009c (45)	Iran	Double-blind	Se: 31 ± 9	Se 200 µg (n = 105)	6 months	①②③	Infertile men with iA and iOAT or idiopathic teratozoospermia of 2 years’ duration
			NAC: 32 ± 10	NAC 600 mg (n = 105)			
			PL: 32 ± 10	PL (n = 106) per day			
Wong 2002 (46)	Netherlands	Double-blind	34.3 ± 3.9	Folic 5 mg (n = 22)	6 months	①②③	Idiopathic infertility, infertility > 2 years, sperm concentration of 5 to 20 million/ml
				Zinc 66 mg (n = 23)			
				PL (n = 106) per day			
Raigani 2014 (47)	Iran	Double-blind	unclear	Folic 5 mg (n = 20)	16 weeks	①②	Infertile men with iOAT, sperm concentrations of <20 × 10^6^/ml, sperm motility <50%, sperm normal morphology < 30%
				Zinc 220 mg (n = 24)			
				PL (n = 25) per day			
Omu 1998 (48)	Kuwait	Open	Zinc: 37.8 ± 7.9	Zinc 500 mg (n = 49)	3 months	④	Infertile men with iA, spermatozoa motility impaired with >40% non-motile sperm
			PL: 38.1 ± 8.2	PL (n = 48) per day			
Omu 2008 (49)	Kuwait	Open	35 ± 1	Zinc 400 mg (n = 11)	3 months	①②	Infertile men with iA, normal sperm concentration (20 to 250 million/ml) but with 40% or more immotile sperm
				PL (n = 8) per day			
Boonyarangkul 2015 (50)	Thailand	Double-blind	Folic: 26.08 ± 0.76	Folic 5 mg (n = 15)	3 months	①②③	Infertile men with iOAT concentration < 15 million/ml, motility < 40%, or morphology < 4%
			PL: 24.7 ± 10.48	PL (n = 15) per day			
Silva 2013 (51)	Brazil	Double-blind	Folic: 35.6 (23-47)	Folic 5 mg (n = 23)	3 months	①②③	Infertility > 2 years, infertile men with iA and iOAT
			PL: 36.8 (24-56)	PL (n = 26) per day			

iA, idiopathic asthenozoospermia; iOA, idiopathic oligoasthenozoospermia; iOAT, idiopathic oligoasthenoteratozoospermia; PL, placebo; LC, l-carnitine; LAC, l-acetylcarnitine; Q10, coenzyme-Q10; C+E, vitamin C+E; ω-3, ω-3 fatty acid; Se, selenium; Folic, folic acid; NAC, N-acetyl-cysteine; RCTs, randomized controlled trials.

① sperm motility; ② sperm concentration; ③ sperm morphology; ④ pregnancy rate.

### Pairwise Meta-Analysis

The results of the pairwise meta-analysis are shown in **Table 2**. We conducted the results of a pairwise meta-analysis that included two or more RCTs. In terms of sperm motility, LC (WMD 8.69% [95% CI: 1.54% to 15.84%]), LC+LAC (WMD 3.77% [95% CI: 1.02% to 6.52%]), Q10 (WMD 4.64% [95% CI: 4.00% to 5.28%]), ω-3 (WMD 4.33% [95% CI: 3.74% to 4.93%]), and Se (WMD 3.32% [95% CI: 2.65% to 3.99%]) had significantly increased sperm motility as compared with placebo. In terms of sperm concentration, the result showed that four antioxidants could improve sperm concentration compared with placebo—LC (WMD 3.65 × 10^6^/ml [95% CI: 2.42 to 4.89 × 10^6^/ml]), Q10 (WMD 5.74 × 10^6^/ml [95% CI: 4.52 to 6.95 × 10^6^/ml]), ω-3 (WMD 10.09 × 10^6^/ml [95% CI: 9.87 to 11.93 × 10^6^/ml]), and Se (WMD 3.91 × 10^6^/ml [95% CI: 2.85 to 5.44 × 10^6^/ml]). In terms of sperm morphology, LC (WMD 3.05% [95% CI: 2.60% to 3.50%]), CoQ10 (WMD 1.74% [95% CI: 1.06% to 2.42%]), and ω-3 (WMD 0.46% [95% CI: 0.22% to 0.71%]) were shown to have treatment effects with statistical significance. In terms of pregnancy rate, 12 studies reported the pregnancy rate, but no significant differences in the pregnancy rate were observed between antioxidants and placebo.

**Table 2:** Pairwise meta-analysis.

Treatment	No. of study	No. of patients (E/C)	WMD (95% CI)	Heterogeneity I^2^ (%)
**Sperm motility**				
LC versus PL	3	61/66	8.69 (1.54, 15.84)	86
LC+LAC versus PL	3	169/91	3.77 (1.02, 6.52)	84
LC versus LC+LAC	1	15/14	−0.93 (−6.86, 5.00)	NA
Q10 versus PL	3	151/150	4.64 (4.00, 5.28)	0
C+E versus PL	2	47/48	−0.44 (−7.76, 6.89)	0
ω-3 versus PL	3	157/146	4.33 (3.74, 4.93)	15
Se versus PL	2	121/124	3.32 (2.65, 3.99)	33
Zinc versus PL	2	47/43	3.00 (−4.62, 10.63)	0
Folic versus PL	4	80/84	0.19 (−1.70, 2.07)	0
Zinc versus Folic	2	42/47	0.14 (−7.71, 7.99)	0
NAC versus PL	1	105/106	1.70 (1.03, 2.07)	NA
NAC versus Se	1	105/105	1.60 (0.85, 2.35)	NA
**Sperm concentration**				
LC versus PL	3	61/66	3.65 (2.42, 4.89)	54
LC+LAC versus PL	2	44/41	0.44 (−4.39, 5.27)	0
LC versus LC+LAC	1	15/14	−1.47 (−13.47, 10.53)	NA
Q10 versus PL	3	151/150	5.74 (4.52, 6.95)	0
C+E versus PL	2	47/48	4.59 (−2.92, 12.09)	0
**ω***-3* versus PL	3	157/146	10.09 (9.87, 11.93)	79
Se versus PL	2	121/124	3.91 (2.38, 5.44)	0
Zinc versus PL	3	58/51	1.23 (−5.00, 7.46)	0
Folic versus PL	3	65/69	2.53 (−3.33, 8.38)	0
Zinc versus Folic	2	42/47	2.63 (−3.48, 8,.74)	0
NAC versus PL	1	105/106	2.90 (1.44, 4.36)	NA
NAC versus Se	1	105/105	1.00 (−0.52, 2.52)	NA
**Sperm morphology**				
LC versus PL	2	35/44	3.05 (2.60, 3.50)	67
LC+LAC versus PL	2	44/41	2.74 (−0.07, 5.56)	79
LC versus LC+LAC	1	15/14	0.47 (−4.01, 4.95)	NA
Q10 versus PL	2	121/120	1.74 (1.06, 2.42)	74
C+E versus PL	2	47/48	−1.37 (−4.58, 1.83)	61
ω-3 versus PL	2	148/137	0.46 (0.22, 0.71)	99
Se versus PL	1	105/106	1.90 (1.16, 2.64)	NA
Zinc versus PL	2	47/43	0.00 (−3.33, 3.33)	0
Folic versus PL	4	80/84	−0.06 (−0.45, 0.32)	0
Zinc versus Folic	2	47/47	−0.72 (−3.69, 2.26)	17
NAC versus PL	1	105/106	1.70 (0.95, 2.45)	NA
NAC versus Se	1	105/105	0.2 (−0.59, 0.99)	NA
**Treatment**	**No. of study**	**No. of patients (E/C)**	**OR (95% CI)**	**Heterogeneity I^2^ (%)**
**Pregnancy rate**				
LC versus PL	2	35/44	0.82 (0.26, 2.58)	0
LC+LAC versus PL	3	56/60	3.11 (0.90, 10.78)	0
LC versus LC+LAC	1	15/14	0.28 (0.04, 1.76)	NA
Q10 versus PL	3	151/150	1.53 (0.43, 5.49)	9
C+E versus PL	1	15/16	NA	NA
LC+LAC versus C+E	1	85/53	3.4 (0.71, 16.17)	NA
Zinc versus PL	1	49/48	4.49 (0.90, 22.35)	NA

PL, placebo; LC, l-carnitine; LAC, l-acetylcarnitine; Q10, coenzyme-Q10; C+E, vitamin C+E; ω-3, ω-3 fatty acid; Se, selenium; Folic, folic acid; NAC, N-acetyl-cysteine; WMD, weighted mean difference; OR, odds ratio; E/C, experimental/control. NA, not applicable.

### Network Meta-Analysis

#### Sperm Motility

**Figure 3:**
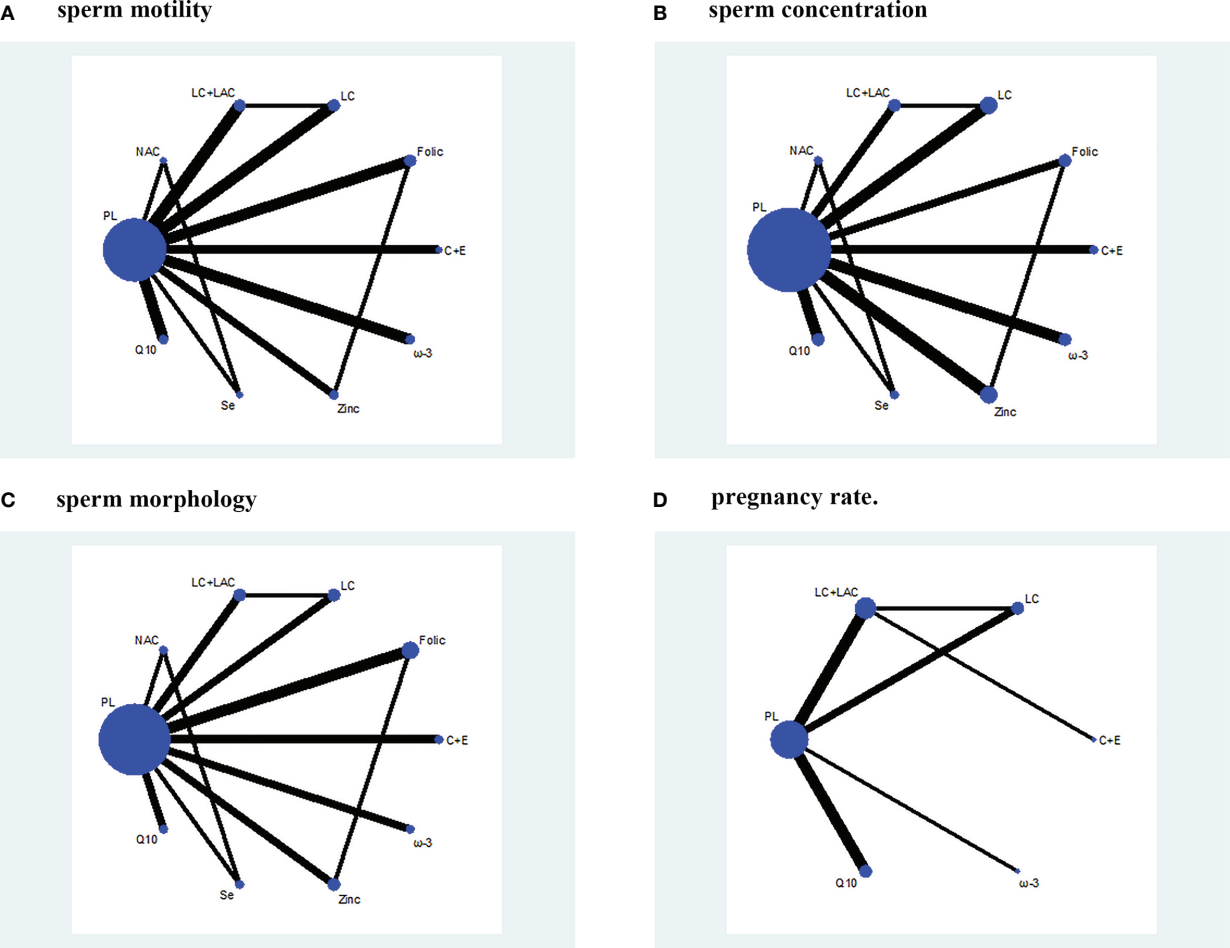
Network plot. **(A)** Sperm motility. **(B)** Sperm concentration. **(C)** Sperm morphology. **(D)** Pregnancy rate. PL, placebo; LC, l-carnitine; LAC, l-acetylcarnitine; Q10, coenzyme-Q10; C+E, vitamin C+E; ω-3, ω-3 fatty acid; Se, selenium; Folic, folic acid; NAC, *N*-acetyl-cysteine.

**Figure 4:**
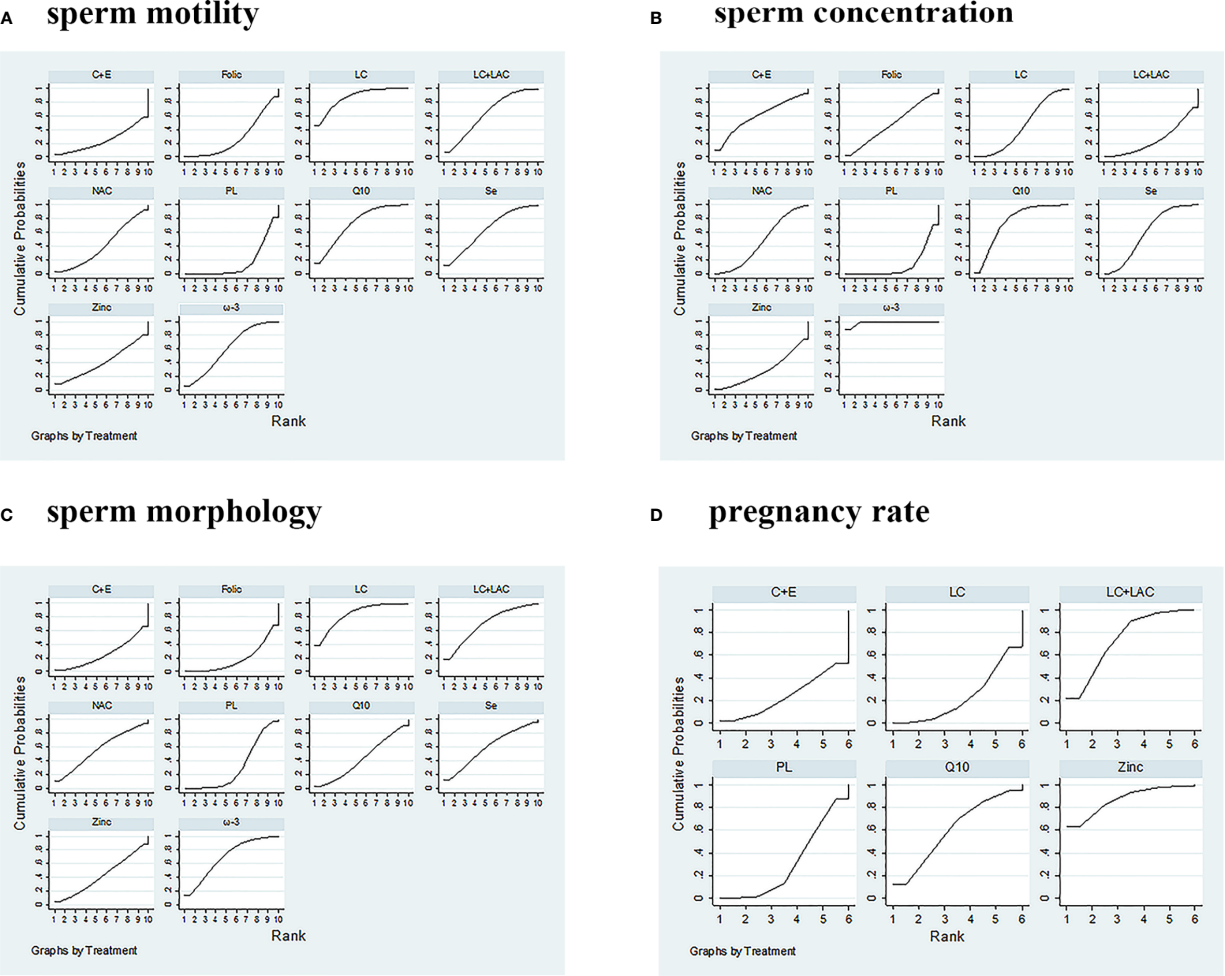
SUCRA ranking curves. **(A)** Sperm motility. **(B)** Sperm concentration. **(C)** Sperm morphology. **(D)** Pregnancy rate. PL, placebo; LC, l-carnitine; LAC, l-acetylcarnitine; Q10, coenzyme-Q10; C+E, vitamin C+E; ω-3, ω-3 fatty acid; Se, selenium; Folic, folic acid; NAC, *N*-acetyl-cysteine; SUCRA, surface under the cumulative ranking.

Sperm motility was reported in 18 RCTs, which included 9 different treatments that led to 27 pairwise comparisons. Compared with placebo, LC (WMD 6.52% [95% CI: 2.55% to 10.50%]), CoQ10 (WMD 4.92% [95% CI: 1.49% to 8.35%]), LC+LAC (WMD 4.21% [95% CI: 0.21% to 8,21%]), ω-3 (4.21% [95% CI: 0.21% to 8.21%]) had significantly increased sperm motility. We did not find other statistically significant differences between the remainder of the active treatments and placebo. Based on SUCRA, the LC had the highest probability of being the most effective treatment to increase sperm motility, followed by CoQ10 and LC+LAC. The network plot is shown in **Figure 3**. The ranking and SUCRA values are shown in **Table 3** and **Figure 4**. Overall results of sperm motility are described in **Table 4**.

**Table 3:** Results of SUCRA values and rank..

Treatment	SUCRA values/rank			
	Sperm motility	Sperm concentration	Sperm morphology	Pregnancy rate
LC	**87.2/2.2**	45.0/5.9	**83.6/2.5**	23.4/4.8
LC+LAC	64.0/4.2	25.3/7.7	70.0/3.7	74.4/2.3
Q10	73.1/3.4	75.6/3.2	43.0/6.1	60.5/3.0
C+E	24.5/7.8	59.8/4.6	25.5/7.7	24.3/4.8
ω-3	61.0/4.5	**98.6/1.1**	69.6/3.7	NA
Se	64.7/4.2	59.9/4.6	59.8/4.6	NA
Zinc	40.4/6.4	29.0/7.4	42.5/6.2	**86.9/1.7**
Folic	28.2/7.5	47.7/5.7	18.1/8.4	NA
NAC	41.0/6.3	46.7/5.8	57.0/4.9	NA
PL	16.0/8.6	12.5/8.9	30.9/7.2	30.6/4.5

Boldface is used to indicate the highest SUCRA values rank.

PL, placebo; LC, l-carnitine; LAC, l-acetylcarnitine; Q10, coenzyme-Q10; C+E, vitamin C+E; ω-3, ω-3 fatty acid; Se, selenium; Folic, folic acid; NAC, N-acetyl-cysteine; SUCRA, surface under the cumulative ranking; NA, not applicable.

**Table 4:** League table of weighted mean difference (WMD) for idiopathic male infertility with sperm motility.

**LC**		-0.93(-6.86,5.00)							**8.69(1.54,15.84)**
1.60 (-3.63,6.84)	**Q10**								**4.64(4.00,5.28)**
2.31 (-2.64,7.26)	0.71 (-4.55,5.96)	**LC+LAC**							**3.77(1.02,6.52)**
3.22 (-3.09,9.53)	1.62 (-4.36,7.60)	0.91 (-5.42,7.24)	**Se**		**1.60(0.85,2.35)**				**3.32(2.65,3.99)**
2.65 (-2.68,7.98)	1.05 (-3.80,5.90)	0.34 (-4.98,5.67)	-0.57 (-6.55,5.41)	**ω-3**					**4.33(3.74,4.93)**
4.82 (-1.49,11.13)	3.22 (-2.76,9.20)	2.51 (-3.82,8.84)	1.60 (-3.31,6.51)	2.17 (-3.81,8.15)	**NAC**				**1.70(1.03,2.07)**
4.71 (-3.93,13.35)	3.11 (-5.33,11.55)	2.40 (-6.26,11.06)	1.49 (-7.65,10.63)	2.06 (-6.39,10.51)	-0.11 (-9.25,9.03)	**Zinc**	0.14(-7.71,7.99)		3.00(-4.62,10.63)
**5.63 (0.05,11.20)**	4.02 (-1.37,9.41)	3.32 (-2.34,8.97)	2.41 (-4.03,8.84)	2.97 (-2.47,8.41)	0.81 (-5.63,7.24)	0.92 (-7.10,8.93)	**Folic**		0.19(-1.70,2.07)
6.88 (-2.14,15.89)	5.27 (-3.53,14.08)	4.56 (-4.47,13.60)	3.65 (-5.82,13.13)	4.22 (-4.59,13.03)	2.05 (-7.42,11.53)	2.16 (-9.03,13.36)	1.25 (-7.86,10.36)	**C+E**	-0.44(-7.76,6.89)
**6.52 (2.55,10.50)**	**4.92 (1.49,8.35)**	**4.21 (0.21,8.21)**	3.30 (-1.60,8.20)	**3.87 (0.44,7.30)**	1.70 (-3.20,6.60)	1.81 (-5.91,9.53)	0.89 (-3.28,5.07)	-0.35 (-8.47,7.76)	**PL**

Bold values indicate statistical signifificance.

The numbers in the upper-right portion of the league table represent the SMDs of total symptom score changes from baseline from the pooling of direct evidence from pairwise meta-analyses. The numbers in the lower-left portion of the league table represent the SMDs of total symptom score changes from baseline from network meta-analysis or indirect evidence. Treatments are arranged in order of the mean ranking from network meta-analysis from the best (left) to the worst (right).

PL, placebo; LC,L-carnitine; LAC,L-acetylcarnitine; Q10,coenzyme-Q10; C+E, Vitamin C+E; ω-3,ω-3 fatty acid; Se, selenium; Folic, folic acid; NAC,N-Acetyl-Cysteine.

#### Sperm Concentration

A summary of 19 RCTs with 9 different treatments was assessed for the sperm concentration. Compared with placebo, ω-3 (WMD 9.89 × 10^6^/ml [95% CI: 7.01 to 12.77 × 10^6^/ml]), Q10 (WMD 5.44 × 10^6^/ml [95% CI: 2.15 to 8.73 × 10^6^/ml]), Se (WMD 3.96 × 10^6^/ml [95% CI: 0.06 to 7.86 × 10^6^/ml]), and LC (WMD 2.75 × 10^6^/ml [95% CI: 0.05 to 5.44 × 10^6^/ml]) had significantly increased sperm concentration (**Table 5**). In comparison, no treatment significantly outperformed the other antioxidants. Based on SUCRA, ω-3 had the highest probability to improve concentration, followed by CoQ10, Se, and LC. The network plot is shown in **Figure 3**. The details as regards ranking and SUCRA values are shown in **Table 3** and **Figure 4**.

**Table 5:** League table of weighted mean difference (WMD) for idiopathic male infertility with sperm concentration.

ω-3									10.09 (9.87, 11.93)
**4.45 (0.10, 8.79)**	**Q10**								**5.74 (4.52, 6.95)**
**5.93 (1.07, 10.78)**	1.48 (−3.63, 6.59)	**Se**			1.00 (−0.52, 2.52)				**3.91 (2.38, 5.44)**
5.33 (−3.11, 13.77)	0.88 (−7.71, 9.48)	−0.60 (−9.44, 8.25)	**C+E**						4.59 (−2.92, 12.09)
8.70 (−1.36, 18.76)	4.26 (−5.93, 14.44)	2.78 (−7.62, 13.17)	3.37 (−9.11, 15.86)	**Folic**			2.63 (−3.48, 8,.74)		2.53 (−3.33, 8.38)
**6.96 (2.10, 11.82)**	2.51 (−2.60, 7.62)	1.03 (−2.90, 4.96)	1.63 (−7.22, 10.48)	−1.75 (−12.14, 8.65)	**NAC**				2.90 (1.44, 4.36)
**7.29 (3.18, 11.39)**	2.84 (−1.59, 7.27)	1.36 (−3.59, 6.31)	1.96 (−6.54, 10.45)	−1.42 (−11.52, 8.69)	0.33 (−4.62, 5.28)	**LC**		−1.47 (−13.47, 10.53)	**3.65 (2.42, 4.89)**
**8.59 (1.45, 15.74)**	4.15 (−3.17, 11.46)	2.67 (−4.93, 10.27)	3.26 (−7.01, 13.54)	−0.11 (−10.69, 10.47)	1.64 (−5.97, 9.24)	1.31 (−5.90, 8.51)	**Zinc**		1.23 (−5.00, 7.46)
**9.08 (2.85, 15.30)**	4.63 (−1.79, 11.05)	3.15 (−3.58, 9.88)	3.75 (−5.91, 13.40)	0.37 (−10.71, 11.46)	2.12 (−4.62, 8.86)	1.79 (−4.36, 7.94)	0.48 (−8.04, 9.01)	**LC+LAC**	0.44 (−4.39, 5.27)
**9.89 (7.01, 12.77)**	**5.44 (2.15, 8.73)**	**3.96 (0.06, 7.86)**	4.56 (−3.38, 12.50)	1.18 (−8.45, 10.82)	2.93 (−0.98, 6.84)	**2.75 (0.05, 5.44)**	1.29 (−5.23, 7.82)	0.81 (−4.68, 6.30)	**PL**

Bold values indicate statistical significance. The numbers in the upper-right portion of the league table represent the SMDs of total symptom score changes from baseline from the pooling of direct evidence from pairwise meta-analyses. The numbers in the lower-left portion of the league table represent the SMDs of total symptom score changes from baseline from network meta-analysis or indirect evidence. Treatments are arranged in order of the mean ranking from network meta-analysis from the best (left) to the worst (right).

PL, placebo; LC, l-carnitine; LAC, l-acetylcarnitine; Q10, coenzyme-Q10; C+E, vitamin C+E; ω-3, ω-3 fatty acid; Se, selenium; Folic, folic acid; NAC, N-acetyl-cysteine.

#### Sperm Morphology

Sperm morphology data were involved in 14 RCTs. In comparison with placebo, only LC (WMD 3.86% [95% CI: 0.91% to 7.53%]) was shown to have treatment effects with statistical significance (**Table 6**). On the other hand, LC was the only treatment that showed significant superiority to another active treatment, folic (WMD 4.96% [95% CI: 0.20% to 9.73%]). Based on SUCRA, LC was ranked the highest over the other antioxidants. The network plot is shown in **Figure 3**. The ranking and SUCRA values are shown in **Table 3** and **Figure 4**.

**Table 6:** League table of weighted mean difference (WMD) for idiopathic male infertility with sperm morphology.

LC	0.47 (−4.01, 4.95)						3.05 (2.60, 3.50)		
1.13 (−3.71, 5.96)	LC+LAC						2.74 (−0.07, 5.56)		
1.34 (−3.57, 6.25)	0.21 (−5.06, 5.48)	**ω-3**					**0.46 (0.22, 0.71)**		
1.96 (−3.96, 7.88)	0.83 (−5.39, 7.05)	0.62 (−5.06, 6.30)	**Se**	0.2 (−0.59, 0.99)			1.90 (1.16, 2.64)		
2.16 (−3.76, 8.08)	1.03 (−5.19, 7.25)	0.82 (−4.86, 6.50)	0.20 (−4.45, 4.85)	**NAC**			1.70 (0.95, 2.45)		
3.23 (−1.88, 8.35)	2.10 (−3.35, 7.56)	1.89 (−2.92, 6.71)	1.27 (−4.56, 7.11)	1.07 (−4.76, 6.91)	**Q10**		**1.74 (1.06, 2.42)**		
3.33 (−2.41, 9.06)	2.20 (−3.84, 8.24)	1.99 (−3.49, 7.47)	1.37 (−5.03, 7.76)	1.17 (−5.23, 7.57)	0.09 (−5.55, 5.74)	**Zinc**	0.00 (−3.33, 3.33)		−0.72 (−3.69, 2.26)
**3.86 (0.19, 7.53)**	2.73 (−1.41, 6.87)	2.52 (−0.75, 5.78)	1.90 (−2.74, 6.54)	1.70 (−2.94, 6.34)	0.63 (−2.91, 4.16)	0.53 (−3.87, 4.93)	**PL**	−1.37 (−4.58, 1.83)	−0.06 (−0.45, 0.32)
4.71 (−1.20, 10.63)	3.58 (−2.64, 9.80)	3.37 (−2.32, 9.07)	2.75 (−3.83, 9.34)	2.55 (−4.03, 9.14)	1.48 (−4.39, 7.35)	1.38 (−5.03, 7.80)	0.85 (−3.81, 5.52)	**C+E**	
**4.96 (0.20, 9.73)**	3.84 (−1.29, 8.96)	3.63 (−0.81, 8.07)	3.01 (−2.52, 8.54)	2.81 (−2.73, 8.34)	1.73 (−2.89, 6.36)	1.64 (−3.19, 6.47)	1.11 (−1.90, 4.11)	0.25 (−5.32, 5.82)	**Folic**

Bold values indicate statistical significance. The numbers in the upper-right portion of the league table represent the SMDs of total symptom score changes from baseline from the pooling of direct evidence from pairwise meta-analyses. The numbers in the lower-left portion of the league table represent the SMDs of total symptom score changes from baseline from network meta-analysis or indirect evidence. Treatments are arranged in order of the mean ranking from network meta-analysis from the best (left) to the worst (right).

PL, placebo; LC, l-carnitine; LAC, l-acetylcarnitine; Q10, coenzyme-Q10; C+E, vitamin C+E; ω-3, ω-3 fatty acid; Se, selenium; Folic, folic acid; NAC, N-acetyl-cysteine.

#### Pregnancy Rate

Although we wanted to find out the effect of antioxidants on pregnancy rate after treatment, only 12 RCTs reported the pregnancy rate, which included 6 different treatments. Despite LC+LAC and CoQ10 having a high ranking in treatment effects as compared with other antioxidants, there is no statistical significance between all antioxidants and placebo (**Table 7**). Furthermore, none of these treatments had shown any statistical superiority to other antioxidants. The network plot is shown in **Figure 3**. The ranking based on SUCRA values in terms of pregnancy rate is shown in **Table 3** and **Figure 4**.

**Table 7:** League table of odds ratio (OR) for idiopathic male infertility with pregnancy rate.

Zinc			4.49 (0.90, 22.35)		
1.79 (0.24, 13.58)	**LC+LAC**		3.11 (0.90, 10.78)	0.28 (0.04, 1.76)	3.4 (0.71, 16.17)
2.38 (0.30, 18.99)	1.33 (0.22, 8.09)	**Q10**	1.53 (0.43, 5.49)		
4.49 (0.90, 22.35)	2.51 (0.73, 8.62)	1.88 (0.51, 7.01)	**PL**	0.82 (0.26, 2.58)	NA
5.46 (0.77, 38.81)	3.05 (0.72, 12.90)	2.29 (0.41, 12.94)	1.22 (0.39, 3.75)	**LC**	
6.09 (0.47, 78.47)	3.40 (0.71, 16.17)	2.55 (0.24, 27.73)	1.36 (0.19, 9.92)	1.12 (0.13, 9.33)	**C+E**

Bold values indicate statistical significance. The numbers in the upper-right portion of the league table represent the SMDs of total symptom score changes from baseline from the pooling of direct evidence from pairwise meta-analyses. The numbers in the lower-left portion of the league table represent the SMDs of total symptom score changes from baseline from network meta-analysis or indirect evidence. Treatments are arranged in order of the mean ranking from network meta-analysis from the best (left) to the worst (right).

PL, placebo; LC, l-carnitine; LAC, l-acetylcarnitine; Q10, coenzyme-Q10; C+E, vitamin C+E; NA, not applicable.

#### The Heterogeneity and Inconsistency

From the results of the meta-analysis, there was some evidence of low-to-moderate statistical heterogeneity on included studies, especially in the outcomes of the sperm motility and sperm concentration (**Table 2**). There are no significant inconsistency factors found in the loops of treatment efficacy (p > 0.05). However, the inconsistency between direct and indirect evidence was identified from the global design-by-treatment interaction model in outcomes of sperm motility (p = 0.043). In the remaining results, we did not find the global inconsistency (p > 0.05) (**Table S2**).

### Publication Bias and Sensitivity Analysis

**Figure 5:**
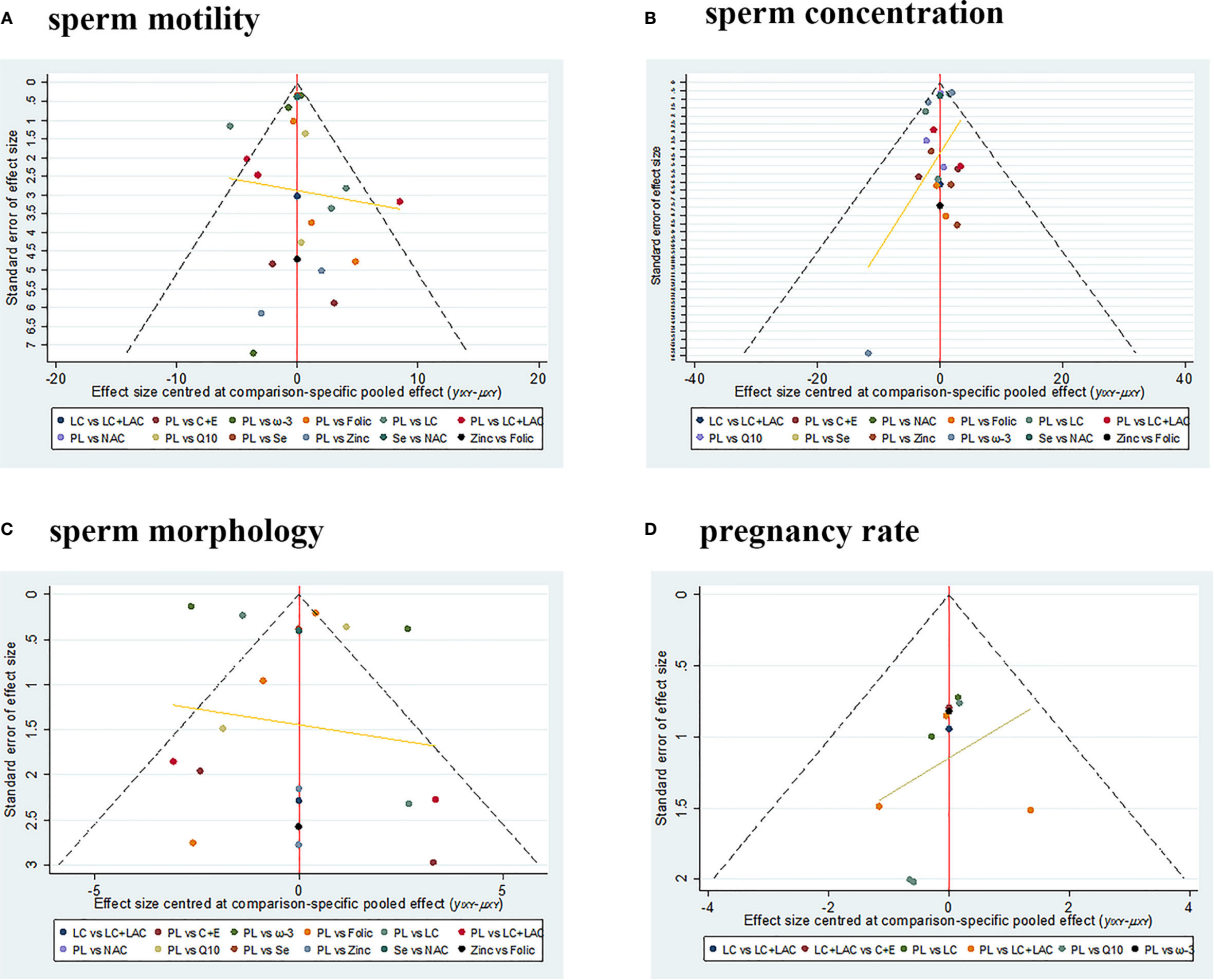
The funnel plots. **(A)** Sperm motility. **(B)** Sperm concentration. **(C)** Sperm morphology. **(D)** Pregnancy rate. PL, placebo; LC, l-carnitine; LAC, l-acetylcarnitine; Q10, coenzyme-Q10; C+E, vitamin C+E; ω-3, ω-3 fatty acid; Se, selenium; Folic, folic acid; NAC, *N*-acetyl-cysteine.

There is no evidence of publication bias in comparison-adjusted funnel plots. The funnel plots are shown in **Figure 5**. In terms of sensitivity, excluding published before 2002 and smaller studies also showed no obvious change in the primary outcomes. So it showed the stability of the results. The results are shown in **Table S3**.

## Discussion

Up until the present, this study is the first study to examine the comparative efficacy of various antioxidants in patients with idiopathic male infertility. Furthermore, the pregnancy rate of patients was also summarized. We found that various antioxidants have different effects on the aspects of sperm parameters.

### Sperm Motility

LC, LC+LCA, CoQ10, and ω-3 showed beneficial effects on sperm motility as compared with placebo. Previous meta-analyses conducted by Buhling (52) and Zhang (53) also demonstrated that carnitine had a favorable effect on sperm motility as compared with placebo. Unlike previous studies’ previous pairwise meta-analyses, our NMA demonstrated that LC was the most effective treatment to increase sperm motility, followed by CoQ10. On the other hand, basic research has been reported that LC and LAC participate in the transportation of long-chain fatty acids to the mitochondrial matrix for β-oxidation, providing energy for β-oxidation of spermatozoa in the epididymis (54). So LC and LAC play a vital role in sperm motility, spermatogenic process, and maturation by enhancing energy metabolism (55). It seems to explain why LC has a better effect on sperm motility. However, some studies had an unclear randomization method and a low number of patients. So the results should be interpreted with caution.

### Sperm Concentration

Compared with placebo, ω-3, CoQ10, Se, and LC had significantly increased sperm concentration. Our NAM demonstrated that ω-3 had the highest probability of being the most effective treatment to increase sperm concentration, followed by CoQ10. Recently, basic research demonstrated that ω-3 fatty acids have anti-inflammatory and antioxidant properties, potentially protecting the composition and function of cell membranes. Furthermore, the successful fertilization of spermatozoa is related to the lipid composition of the spermatozoa membrane (56, 57). Consistent with this finding, the previous meta-analysis also showed that ω-3 fatty acids had a positive effect on sperm concentration compared with placebo (58). However, there are few reports on the influence of ω-3 on pregnancy rate, and no major side effects were reported from the included studies. So more RCTs conducted in large samples of participants are needed to verify the therapeutic effects of ω-3 on pregnancy rate and side effects.

### Sperm Morphology

The NMA demonstrated that CoQ10 and ω-3 were near the critical statistical significance as compared with placebo. But only LC was shown to have treatment effects with statistical significance. Contrastingly, our pairwise meta-analysis demonstrated that ω-3 and CoQ10 were shown to have treatment effects with statistical significance. The previous meta-analysis conducted by Salas (59) also demonstrated that ω-3 and CoQ10 had a favorable effect on sperm morphology as compared with placebo. The controversy of this result might be related to the lack of study and the differences in data after mixed comparison in the NMA. So we need more RCTs to verify the therapeutic effects on the morphology of ω-3 and CoQ10. However, there is no doubt that LC has the best therapeutic effect on sperm morphology.

### Pregnancy Rate

The most objective outcome to show the effect of treatment on male fertility must be the pregnancy rate. However, only 12 RCTs reported the pregnancy rate. There is no statistical significance between treatment and placebo. Most studies have reported effects on sperm parameters but have not described the pregnancy rate in detail. On the other hand, the shorter follow-up time would result in lower positive rates. We must point out that “fertility” also depends on the fertility status of the female partner. It would also clearly influence the outcome of medical intervention in the male partner, although the previous meta-analysis showed that supplementation with antioxidants has a positive effect on pregnancy rate as compared with placebo (60). However, due to the lack of data in each study, the results are not applicable to idiopathic male infertility.

Although CoQ10 was not ranked first in four aspects of sperm parameters in this study, CoQ10 has an obvious therapeutic effect on sperm motility and sperm concentration. A previous meta-analysis study also concludes that CoQ10 has a profound effect on sperm motility and concentration (61). In the electron-transport system, CoQ10 is an antioxidant molecule that plays a vital role, and CoQ10 could inhibit the formation of organic peroxides in sperm to reduce sperm cell OS (62, 63). Based on our result, CoQ10 could be a good choice for idiopathic male infertility.

It is worth emphasizing that previous RCTs and meta-analyses did not analyze the optimal dosage and medication time. Until now, the exact dosages and regimens have not been clearly defined (64). Recently, a study has shown that excessive intake of antioxidants can change the oxidation–reduction equilibrium into reductive stress, and it has the same adverse effect on sperm quality as OS (65). In our study, the follow-up period was from 3 to 8 months, and the dosage of the same type of antioxidants was roughly the same. Compared with placebo, some antioxidants such as zinc, vitamin E+vitamin C, and folic were of no statistical significance. But we do not know whether the reductive stress was caused by a high amount/dose of antioxidants or lack of follow-up time. On the other hand, some studies have reported that hormones and SERMs increased sperm quality (66). We need more experimental studies on these interventions with significant effect sizes so that a better treatment could be planned to improve the outcome.

### Limitations and Future Studies

Several limitations of our NMA should be stated. The internal effectiveness of NMA mainly relies on the realization of the required assumptions, like heterogeneity, inconsistency, and transitivity (67). First, although the results of inconsistency factors were not found in the loops of treatment efficacy, the global inconsistency was existing in the outcome of sperm motility. Second, we found that some treatments had high statistical heterogeneity based on I^2^. It could be caused by methodological heterogeneity (clinical measurement tool and point of evaluation) and clinical heterogeneity (age, duration of infertility, or dosage). So considering the heterogeneity between studies, we used the random-effects model to pool the pairwise estimates. Third, many treatment pairs include two studies or at the most a maximum of three studies, which were not sufficient to correctly assess the transitivity assumption. So we are looking forward to getting more clinical data in the future. Furthermore, the applicability of our findings might be limited, because the NMA was based on indirect comparisons but not direct comparisons. At last, we have included the main outcomes of sperm motility, sperm morphology, sperm concentration, and pregnancy rate. Because of the lack of data in each study, we could not include some outcomes like forwarding sperm motility and sperm volume. Therefore, we have to be cautious when evaluating the outcomes of different treatments.

We also hope that this article will give some enlightenment to the focus in our future research. First of all, some RCTs have gradually begun to use combinations of different types of antioxidants, for example, using the mixture of selenium+zinc+vitamin E or LC+CoQ10+vitamin C+zinc (68, 69). Those combinations were also more efficacious than placebo in sperm quality parameters. However, due to the lack of research literature and difficulty in distinguishing the effects of combinations of various antioxidants, those studies were not included. Therefore, in the future, we need more RCTs to verify the therapeutic effects of combining different types of antioxidants.

## Conclusion

Our results suggest that compared with other antioxidants, l-carnitine had the highest probability of being the most effective treatment to increase sperm motility and morphology. ω-3 fatty acids have the most positive effect on sperm concentration. Coenzyme-Q10 is a better effective treatment for sperm motility and concentration. In terms of pregnancy rate, there is no statistical significance between treatment and placebo, and further evidence is awaited to verify the effect of antioxidants on the pregnancy rate.

## Data Availability Statement

The original contributions presented in the study are included in the article/**Supplementary Material**. Further inquiries can be directed to the corresponding author.

## Author Contributions

K-pL: protocol development, data collection and management, data analysis, and manuscript writing. X-sY: protocol development, data collection and management, data analysis, and manuscript writing. TW: protocol development, data management, data analysis, and manuscript writing.

## Funding

This work was supported by the City of Nanchong Strategic Cooperation with Local Universities Foundation of technology (20SXQT0305); the Application and Basic Research Program of the Sichuan Science and Technology Department (2020YJ0185); and The Primary Health Development Research Center of Sichuan Province Program (SWFZ21-C-98).

## Conflict of Interest

The authors declare that the research was conducted in the absence of any commercial or financial relationships that could be construed as a potential conflict of interest.

## Publisher’s Note

All claims expressed in this article are solely those of the authors and do not necessarily represent those of their affiliated organizations, or those of the publisher, the editors and the reviewers. Any product that may be evaluated in this article, or claim that may be made by its manufacturer, is not guaranteed or endorsed by the publisher.
