# Supplementary material for: The Effect of Antioxidants on Sperm Quality Parameters and Pregnancy Rates for Idiopathic Male Infertility: A Network Meta-Analysis of Randomized Controlled Trials
Source: Front Endocrinol (Lausanne). 2022 Feb 21;13:810242. doi: 10.3389/fendo.2022.810242 (PMC8898892; doi:10.3389/fendo.2022.810242)
Supplement: Supplementary file 1 [file DataSheet_1.docx]

| **Table S1 Searching strategies** | |
| --- | --- |
| step | Search algorithm |
| #1 | male infertility |
| #2 | idiopathic infertility |
| #3 | idiopathic male infertility |
| #4 | asthenozoospermia |
| #5 | idiopathic asthenozoospermia |
| #6 | oligoasthenozoospermia |
| #7 | idiopathic oligoasthenozoospermia |
| #8 | oligoasthenoteratozoospermia |
| #9 | idiopathic oligoasthenoteratozoospermia |
| #10 | subfertile |
| #11 | idiopathic subfertile |
| #12(patient) | #1 OR #2 OR #3 OR #4 OR #5 OR #6 or #7 OR #8 OR #9 OR #10 OR #11 |
| #13 | antioxidants |
| #14 | nutrients |
| #15 | mineral |
| #16 | L-carnitine |
| #17 | L-acetylcarnitine |
| #18 | coenzyme-Q10 |
| #19 | *ω*-3 fatty acid |
| #20 | selenium |
| #21 | zinc |
| #22 | Vitamin E |
| #23 | Vitamin C |
| #24 | folic |
| #25 | N-Acetyl-Cysteine |
| #26(intervention) | #13 OR #14 OR #15 OR #16 OR #17 OR #18 OR #19 OR #20 OR #21 OR #22 OR #23 OR #24 OR #25 |
| #27(Comparator) | placebo |
| #28(I+C) | #12 OR #26 |
| #29 | semen quality Parameters |
| #30 | semen quality |
| #31 | Semen Parameters |
| #32 | sperm motility |
| #33 | sperm concentration |
| #34 | sperm morphology |
| #35 | pregnancy rate |
| #36(outcomes) | #29 OR #30 OR #31 OR #32 OR #33 OR #34 OR #35 |
| #37(PICO) | #12 and #28 and #36 |
| #38(RCT) | randomized |
| #39 | randomized trial |
| #40 | randomized controlled trial |
| #41 | #38 OR #39 OR #40 |
| Search | #37 and #41 |

| **Table S2 Global inconsistency in networks** | | | |
| --- | --- | --- | --- |
| **outcomes** | **χ^2^** | | ***P* value** |
| sperm motility | 15.21 | | 0.043 |
| Sperm concentration | 0.67 | | 0.98 |
| sperm morphology | 2.55 | | 0.6364 |
| pregnancy rate | 0.13 | | 0.9373 |
| **Loop inconsistency in networks** | | | |
| **Closed triangular loop of evidence** | **Inconsistency factor (95% confidence interval)** | ***P* value** | **Loop heterogeneity t^2^** |
| **sperm motility** |  |  |  |
| PL-LC-LC+LAC | (0.00,20.32) | 0.782 | 31.015 |
| PL-Zinc-Folic | (0.00,15.16) | 0.625 | 0.000 |
| **Sperm concentration** |  |  |  |
| PL-LC-LC+LAC | (0.00,17.70) | 0.478 | 0.000 |
| PL-Se-NAC | (0.00-25.40) | 0.853 | 0.000 |
| PL-Zinc-Folic | (0.00,20.35) | 0.879 | 0.000 |
| **sperm morphology** |  |  |  |
| PL-LC-LC+LAC | (0.00,8.17) | 0.418 | 0.000 |
| PL-Zinc-Folic | (0.00,9.00) | 0.341 | 0.000 |
| **pregnancy rate** |  |  |  |
| PL-LC-LC+LAC | (0.00,3.18) | 0.911 | 0.000 |
| PL, placebo; LC, L-carnitine; LAC, L-acetylcarnitine; Folic, folic acid; NAC, N-Acetyl-Cysteine | | | |

| **Table S3 SUCRA rank of treatment for overall and sensitivity analysis** | | | | |
| --- | --- | --- | --- | --- |
| **treatment** | **overall** | **Excluding studies with**  **high risk of bias** | **Excluding studies sample**  **size <30** | **Excluding studies**  **published before 2002** |
| **sperm motility** |  |  |  |  |
| LC | 2.2 | 3.2 | 2.4 | 2.3 |
| LC+LAC | 4.2 | 4.4 | 4.5 | 4.3 |
| Q10 | 3.4 | 3.2 | 3.8 | 3.7 |
| C+E | 7.8 | 7.8 | 7.8 | 5.7 |
| ω-3 | 4.5 | 4.3 | 4.7 | 4.5 |
| Se | 4.2 | 5.0 | 4.4 | 5.2 |
| Zinc | 6.4 | 5.8 | 6.0 | 6.4 |
| Folic | 7.5 | 5.8 | 6.1 | 7.5 |
| NAC | 6.3 | 6.8 | 6.5 | 6.8 |
| PL | 8.6 | 8.6 | 8.7 | 8.6 |
| **Sperm**  **concentration** |  |  |  |  |
| LC | 5.9 | 4.9 | 5.8 | 6.0 |
| LC+LAC | 7.7 | 7.4 | 7.4 | 7.5 |
| Q10 | 3.2 | 3.4 | 3.4 | 3.6 |
| C+E | 4.6 | 4.6 | 4.6 | 3.3 |
| ω-3 | 1.1 | 1.2 | 1.2 | 1.4 |
| Se | 4.6 | 4.8 | 4.6 | 4.8 |
| Zinc | 7.4 | 7.3 | 7.1 | 7.0 |
| Folic | 5.7 | 6.9 | 6.8 | 6.8 |
| NAC | 5.8 | 5.8 | 5.6 | 5.8 |
| PL | 8.9 | 8.6 | 8.5 | 8.6 |
| **Sperm morphology** |  |  |  |  |
| LC | 2.5 | 2.5 | 2.5 | 2.4 |
| LC+LAC | 3.7 | 3.7 | 3.7 | 3.6 |
| Q10 | 6.1 | 6.1 | 6.1 | 6.0 |
| C+E | 7.7 | 7.4 | 7.5 | 8.9 |
| ω-3 | 3.7 | 3.8 | 3.8 | 3.7 |
| Se | 4.6 | 4.6 | 4.6 | 4.5 |
| Zinc | 6.2 | 6.4 | 6.3 | 6.0 |
| Folic | 8.4 | 8.7 | 8.7 | 8.1 |
| NAC | 4.9 | 4.8 | 4.9 | 4.7 |
| PL | 7.2 | 7.0 | 7.0 | 7.0 |
| **pregnancy rate** |  |  |  |  |
| LC | 4.8 | 3.8 | NA | 3.8 |
| LC+LAC | 2.3 | 1.6 | NA | 1.6 |
| Q10 | 3.0 | 2.2 | NA | 2.2 |
| C+E | 4.8 | 3.9 | NA | 3.9 |
| ZINC | 1.7 | NA | NA | NA |
| PL | 4.5 | 3.5 | NA | 3.5 |
| PL, placebo; LC, L-carnitine; LAC, L-acetylcarnitine; Q10, coenzyme-Q10; C+E, Vitamin C+E; *ω*-3, *ω*-3 fatty acid; Se, selenium; Folic, folic acid; NAC, N-Acetyl-Cysteine; | | | | |
